# The Understanding of Ebola Virus Disease (EVD) Among Medical Practitioners of Karachi, Pakistan

**DOI:** 10.3390/tropicalmed2020016

**Published:** 2017-06-14

**Authors:** Sadia Shakeel, Wajiha Iffat, Atta Abbas Naqvi, Fouzia Ahmed, Shugufta Usmani, Manahil Mazhar, Ayesha Nisar

**Affiliations:** 1 Dow College of Pharmacy, Dow University of Health Sciences, Karachi 75270, Pakistan; sadia.shakeel@duhs.edu.pk (S.S.); wajiha.iffat@duhs.edu.pk (W.I.); manahil_mazhar@hotmail.com (M.M.); ayesha.nisar@live.com (A.N.); 2 Department of Pharmacy Practice, College of Clinical Pharmacy, Imam Abdulrahman Bin Faisal University (University of Dammam), Dammam 31441, Eastern Province, Saudi Arabia; 3 Jinnah University for Women, V – C, Nazimabad, Karachi 74600, Pakistan; fouzia.ahmed01@gmail.com; 4 Faculty of Pharmacy, Hamdard University, Sharae Madinat al-Hikmah, Mohammad Bin Qasim Avenue, Karachi 74600, Pakistan; shagufta_usmani@hotmail.com

**Keywords:** Ebola virus disease, healthcare practitioners, Pakistan

## Abstract

The World Health Organization (WHO) has acknowledged the large West African Ebola virus disease (EVD) outbreak to be a community health disaster of global concern, and the spread of disease demands a synchronized response. Medical practitioners have an increased risk of contracting the disease as compared to others as they are directly exposed to patients’ blood or fluids. This study evaluated the knowledge of medical practitioners in Karachi regarding EVD. It was descriptive and exploratory in nature and took place over a period of 4 months, i.e., August 2016 to November 2016. The respondents were randomly selected by convenience sampling and surveyed with a 20-item questionnaire. Overall, 403 questionnaires were included in the study and a response rate of 80.6% was achieved. The majority (56.3%) considered themselves to be somewhat knowledgeable; females had more knowledge as compared to male (*p* < 0.003). More than 80% knew about the 2014 Ebola outbreak in West Africa. Interestingly, the findings revealed that respondents’ knowledge about diagnosis and identification of EVD is good. Respondents considered EVD a severe disease and emphasized on the need for protective measures when contacting affected patients. Interventions should be tailored to focus on areas where respondents showed a lack of knowledge about the disease.

## 1. Introduction

Ebola virus disease (EVD) is a potentially fatal disease caused by Ebola virus. It results in viral hemorrhagic fever characterized by internal hemorrhage. It has high mortality rates, as it was observed to cause fatalities in 90% of the infected patients. The high fatality rate, coupled with the lack of treatment options and vaccination, renders Ebola a threat to the community at large [1]. EVD virus results in acute febrile hemorrhagic illness exhibiting several clinical manifestations ranging from fever and fatigue to headaches, vomiting, violent diarrhea, and, subsequently, multiple organ failure and massive internal bleeding [2].

EVD is caused by Ebola virus species in humans and nonhuman primates (gorillas, monkeys, and chimpanzees) [3]. EVD belongs to the family *Filoviridae* and the genus *Ebolavirus*, and has five recognized species, among which the first four can cause infections in humans, namely *Zaire ebolavirus*, *Sudan ebolavirus*, *Taï Forest ebolavirus*, *Bundibugyo ebolavirus*, and *Reston ebolavirus* [4]. The virus was first discovered in 1976 when two unrelated Ebola hemorrhagic fever (EHF) outbreaks occurred in northern Zaire (Yambuku) and southern Sudan [5]. Since 1976, there has been a total of 885,343 suspected and laboratory confirmed cases of EHF reported in West Africa, making this part of the world persistently exposed to the disease [6].

Ebola virus infects the host through abrasions in the skin, mucosal surfaces, or by parenteral means. It is considered that Ebola spreads via human-to-human transmission by means of direct contact with the secretions, blood, organs, or other bodily fluids of infected persons and with surfaces and materials (e.g., bedding, clothing) polluted with these fluids. The majority of human infections in outbreaks seems to occur by direct contact with infected patients or cadavers [7]. Transmission in health care settings has found to be associated frequently with EHF outbreaks in Africa [8]. The Ebola outbreak in West Africa that started in the month of March 2014 was the most lethal epidemic since the Ebola virus was first recognized in 1976. The number of cases and mortality reported in the outbreak exceeded all previously reported mortality due to EVD [9,10,11]. The public health significance of EHF lies in not only its potential to cause significant mortality and morbidity but also its potential for nosocomial spread. As an extremely communicable disease, EVD can spread to other parts of the world due to the fact that people are continuously moving.

In response to the 2014 outbreak, Pakistan took measures to limit the spread of infection by setting up screening facilities at all airports for passengers entering the country from Africa [12]. Furthermore, health authorities initiated workshops and education programs for doctors [13]. Medical practitioners had the highest risk of contagion compared to others, as they were in a position to come into direct contact with patients’ blood or fluids. The use of personal protective equipment (PPE) is recommended by WHO. Efforts were made by the health care authorities to issue guiding principles concerning the personal protective practices of healthcare professionals and clinical case management of EVD, including approaches toward EVD treatment and prevention in hospital settings.

Although Ebola is not prevalent in Pakistan, it is still recognized as an epidemic threat for the country [13]. The health authorities of Pakistan are preparing the workforce by conducting training for healthcare practitioners (HCPs), along with prevention strategies, in the event an Ebola emergency occurs. This study was conducted to assess the knowledge and attitudes of HCPs towards EVD in Pakistan and to find out if educational training has increased their knowledge with regards to countering the threat.

## 2. Methods

### 2.1. Study Design and Duration

This was a descriptive, cross-sectional exploratory study conducted for a period of 4 months, i.e., August 2016 to November 2016, to evaluate knowledge of EVD in HCPs of Karachi, Pakistan.

### 2.2. Study Population and Eligibility Criteria

All medical practitioners, i.e., doctors registered with Pakistan Medical and Dental Council (PM&DC), were identified as target population. Eligibility criteria included doctors practicing medicine at public sector, as well as private clinical and hospital settings in the city of Karachi, Pakistan. Those belonging to other fields of health care as well as those who did not consent to participate, and incomplete responses, were not included our study.

### 2.3. Sample Size

According to PM&DC records, a total of 71,109 doctors was registered in the city of Karachi, Pakistan [14]. This figure was assumed as the total population for our study. The sample size was calculated using online software for sample size calculation (Raosoft Inc.^®^, Seattle, DC, USA). The required sample size calculation was 377.

### 2.4. Sampling Technique

The participants were randomly selected by convenience sampling from the record of general practitioners, Karachi branch, provided by the Forum of General Medical Practitioners (FGMP). Practitioners were approached in their free time.

### 2.5. Research Instrument Development and Piloting

A questionnaire was developed by reviewing the available literature on the topic. The questionnaire contained 8 questions related to demographic information and 20 questions related to knowledge of the respondents. The piloting of the questionnaire was carried out on 30 HCPs and the questionnaire was validated. The Kaiser-Meyer-Olkin (KMO) measure of sampling adequacy reported a value of 0.654, and Bartlett’s test of sphericity was significant, i.e., *p* < 0.0001.

### 2.6. Data Collection and Analysis

The questionnaires were left with the respondents for a week. Following this time period, the filled questionnaires were collected. The questionnaire items were analyzed with IBM SPSS version 20 (Statistical Package for Social Sciences, IBM Corporation, Armonk, New York, NY, USA). Descriptive statistics and frequency were used to demonstrate respondents’ demographic information and responses. Pearson’s chi-square (X^2^) test was employed for evaluating the relationship between independent variables (demographic) and dependent variable (knowledge) of respondents. A value of *p* < 0.05 was considered significant.

### 2.7. Statement of Consent and Ethical Consideration

Prior to the data collection, the respondents were briefed about the aims and objectives of the study, and their consent was obtained. The involvement in the research was voluntary with secrecy. The respondents were neither pressurized nor incentivized for their participation. The study was exempted from ethical review.

## 3. Results

Out of 500 questionnaires, 410 questionnaires were returned. Seven questionnaires were found uncompleted and were not included. A total of 403 questionnaires was incorporated in the present study with a response rate of 80.6%.

### 3.1. Demographic Information:

Out of 403 respondents, 42.92% were male while 57.07% respondents were female. Half of the respondents were in the age range of 25–30 years (50.86%). Most of them (50.86%) were between 25 and 30 years and were practicing in public sector health care settings (55.32%). Half of the practitioners (49%) were in faculties of medical colleges. Almost a quarter of them (22.08%) were general practitioners. The detailed demographic characteristics and practice information are shown in Table 1.

### 3.2. Knowledge Regarding EVD

Only a small segment (3.9%) of respondents considered themselves to be very knowledgeable. The majority (56.3%) considered themselves to be somewhat knowledgeable; females had more knowledge as compared to males (*p* = 0.017). The status of knowledge was also significantly associated with the experience (*p* = 0.001) and designation (*p* = 0.001) of respondents. Mass media (47.6%) and medical literature (37.7%) were found to be the major source of information for EVD. More than 80% knew about the 2014 Ebola outbreak in West Africa. The results are summarized in Table 2.

### 3.3. Knowledge about Transmission and Source of Infection

**Figure 1:**
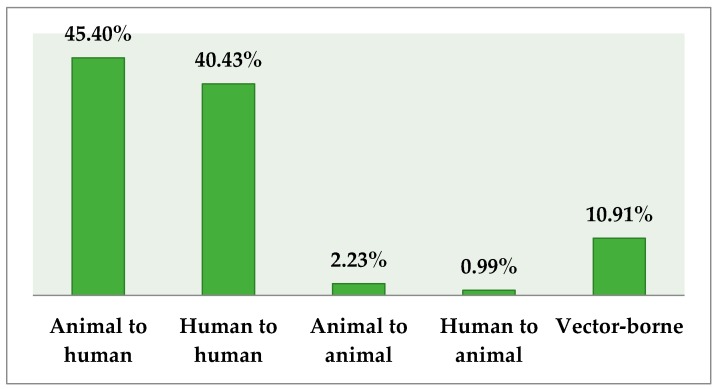
Respondents’ knowledge regarding route of transmission of Ebola virus disease (EVD) (%).

Animal-to-human (45%) and human-to-human (41%) interactions were believed to be the route of transmission of EVD. Figure 1 highlights the findings.

**Figure 2:**
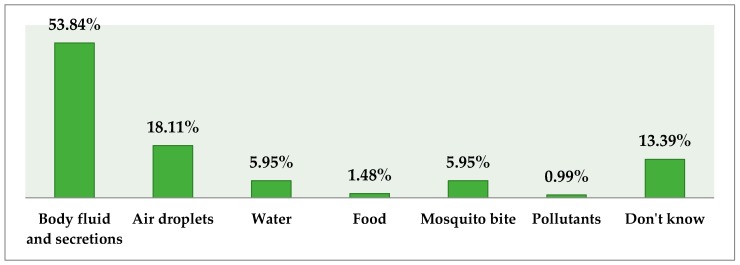
Respondents’ beliefs regarding source of EVD infection (%).

Body fluid and secretions were believed to be the major sources of EVD infection as highlighted by 54% and 18% of respondents, respectively. Only a small proportion (6%) considered water and mosquitoes as an infection source. A significant association was reported between the responses and the amount of experience as well as the age of respondents (*p* < 0.0001). More than half (53%) of respondents were aware of the existence of reservoirs of EVD and slightly more than half (51.6%) considered fruit bats as the major reservoir; other reservoirs as highlighted by doctors (with proportions given here as percentage values) included mosquitoes (16.4%), cattle (14.1%), and pigs (10.9%). Knowledge of respondents regarding reservoirs of infection was significantly associated with the age and experience of the respondents (*p* < 0.0001). Nearly half of the medical practitioners (45%) highlighted that the incubation period of the virus is between 2 and 21 days. The female practitioners were more familiar with the incubation period as compared to their male counterparts (*p* = 0.017). Respondents’ beliefs regarding the source of infection are depicted in Figure 2.

### 3.4. Knowledge about Clinical Features of EVD

**Figure 3:**
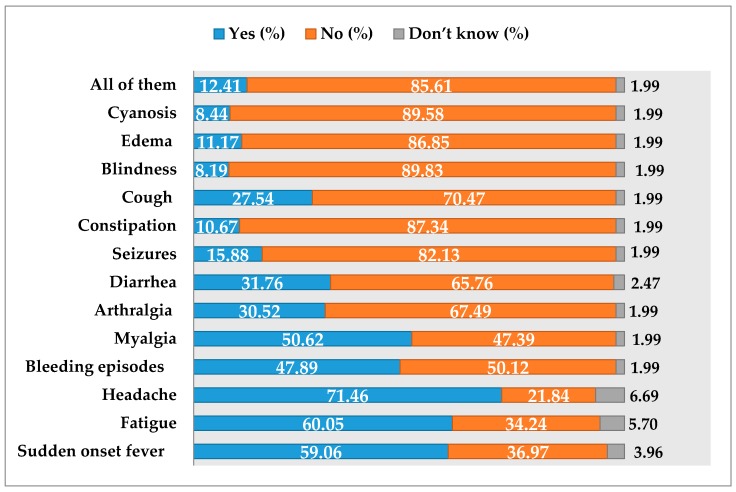
Respondents’ beliefs about symptoms of EVD.

Headache (71.46%), fatigue (60.05%), sudden onset fever (59.06%), myalgia (50.62%), and bleeding episodes (47.89%) were the commonly known clinical features of EVD as highlighted by the respective proportions of practitioners. Most common measures to contain EVD outbreak, as reported by respondents, included isolation and quarantine (63.28%), proper handling and disposal of dead bodies (46.65%), prompt case management (41.19%), mass vaccination (29.78%), and mosquito control (21.59%). Nearly half of the respondents (44.1%) considered the required quarantine period to be 3 weeks, while a third (32.6%) considered that period to be 1 week. Similarly, a sizeable percentage (43%) of respondents knew the role of isolation post-recovery. Their beliefs about clinical features of EVD are illustrated in Figure 3.

### 3.5. Respondents’ Opinion about the Management Strategies

Regarding the available case management strategies, more than half of the practitioners (66.93%) believed that supportive treatment was the best management strategy. The majority of the target segment (70%) agreed that hand hygiene, personal protective equipment (PPE), and proper handling of needles play a role in personal protection. An overwhelming majority (81%) agreed that PPE recommended by WHO included double examination gloves, a medical mask or face shield, an impermeable gown, goggles, and shoe and head covers have the potential to protect users of such PPE from contracting EVD infection. The results are summarized in Table 3.

### 3.6. Knowledge about Diagnosis of EVD

**Figure 4:**
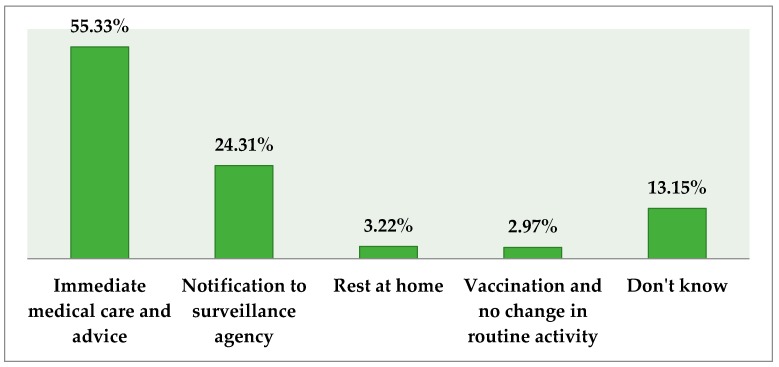
Respondents’ action in case of suspicion of EVD.

On questioning the respondents’ action in case of suspicion of EVD, slightly more than half of the target segment (56%) believed that they would provide immediate medical care and advice, and a quarter (25%) mentioned notifying the surveillance agencies. Refer to Figure 4 for details.

**Figure 5:**
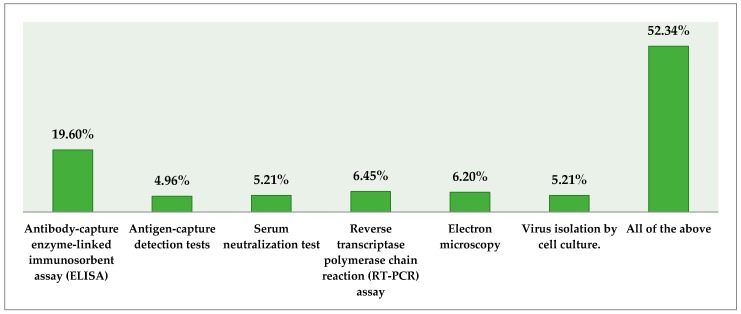
Respondents’ opinion regarding diagnostic tools for EVD.

Slightly more than half (52%) of practitioners agreed that antigen-capture detection tests, electron microscopy, serum neutralization tests, antibody-capture enzyme-linked immunosorbent assays (ELISA), reverse transcriptase polymerase chain reaction (RT-PCR) assays, and virus isolation by cell culture could be used for investigating EVD. Respondents’ opinions regarding diagnostic tools for EVD are illustrated in Figure 5.

## 4. Discussion

A novel study was conducted in Pakistan to investigate the understanding of medical practitioners towards EVD. Only a small proportion (3.9%) of respondents considered themselves very knowledgeable about EVD, whereas slightly more than half of respondents (56.3%) considered themselves somewhat knowledgeable. This finding highlights the need for necessary measures to improve the level of knowledge of doctors by execution of educational interventions such as continuous medical education (CME). Additionally, effective training of staff and implementation of standard operating procedures can also be effective approaches in view of the risk of EVD to practitioners. These strategies are also supported by Kilmarx et al., in their report on EVD among health care workers (HCWs) in 2014 [15].

We found that an overwhelming majority of practitioners; i.e., more than 80% knew about the 2014 Ebola outbreak in West Africa. This was encouraging, as a similar study conducted among HCWs in India reported that a high percentage was unaware of the phenomenon [16]. Mass media (47.6%) and medical literature (37.7%) were the most common sources of information for EVD. In congruence, a study conducted in India reported that radio and television (TV) were the most common source of knowledge about EVD [16]. In this study, respondents’ knowledge about the symptoms and diagnosis of EVD was considerably better compared to that of other aspects of the disease. However, this finding contradicts that of Matta et al., who reported that the knowledge of HCWs regarding symptoms was well below par [17]. This divergence could be a result of the fact that more importance is given to the symptoms of EVD in awareness programs than on other areas. This is emphasized by Dunklin et al., who stated that the recognition of symptoms was necessary for early diagnosis and management of EVD [18].

Individuals are infected by EVD by coming in close contact with blood, secretions, organs, or other body fluids of infected animals, such as fruit bats, monkeys, chimpanzees, gorillas, forest antelopes, and porcupines, found ill or dead or in the tropical forest [19,20]. In this study, animal-to-human contact was mentioned by nearly half of the doctors (45%), and human-to-human contact, also highlighted by a high proportion (41%), were thought to be the most common route of transmission of EVD. Body fluids and secretions were mentioned by slightly more than half (54%) of practitioners whereas only a small percentage of respondents (6%) considered water and mosquitoes as a potential source of infection. Interestingly, the findings revealed that respondents’ knowledge about diagnosis and identification of EVD is good; more than half (52%) agreed that an antibody-capture enzyme-linked immunosorbent assay (ELISA), a serum neutralization test, antigen-capture detection tests, electron microscopy, a reverse transcriptase polymerase chain reaction (RT-PCR) assay, and virus isolation by cell culture could be used to investigate EVD. It is pertinent to mention that practitioners with work experience of more than 10 years appeared well-informed of EVD in comparison to those with work experience of less than 3 years. This finding lines up with other studies that stated better awareness of experienced HCWs [21]. The probable cause for this direct relationship of increasing knowledge with experience could be due to executive positions being held by experienced practitioners that allow them to participate in continuous medical education (CME) activities that improve their knowledge over time. This assumption may also be supported by a report that indicated that experienced workers are more efficient in dealing with patients in healthcare settings [22].

Our findings indicate that health practitioners considered EVD a severe disease and emphasized the need of employing protective measures during contact with affected patients. More than half of the target segment, i.e., around 70%, agreed that hand hygiene, personal protective equipment (PPE), and the proper handling of needles play a role in personal protection. The majority (81%) agreed that PPE recommended by WHO, including double examination gloves, a medical mask/face shield, an impermeable gown, goggles, and shoe and head covers, can limit the chance of contracting an infection. Another study reported that HCWs had positive attitudes towards protective measures but that they failed to transform them into practice [23]. Pakistan is already an under-resourced state combating other infectious diseases such as polio, measles, and dengue. Additional disease burden on the health care system with EVD would be detrimental to not only the patients but the health authorities as well. Hence, by educating the doctors, policy-makers, paramedical staff, and community about the protective measures, the risk of an Ebola outbreak can be avoided. This plan was successfully executed in Pakistan during the Ebola outbreak in West Africa [13].

The limitations of the present study include the restriction to the population of a small number of institutions in Karachi. As with every retrospective study, our study faced the probable influence of issues with recall and social desirability response bias. Further limitations consist in the scarcity of research studies regarding EVD, thus making it difficult to generalize the findings in Pakistan. One of the major limitations was the reporting of self-perceived knowledge level by the respondents. The authors recommend developing an instrument to document knowledge of the subject in this population in order to have more thorough understanding of knowledge level.

## 5. Conclusions

The knowledge of medical practitioners about EVD was sub-optimal. While further studies should be conducted on a national scale to authenticate these results, interventions should be tailored to focus areas where respondents showed a lack of knowledge about the disease. Such an educational intervention may be the most successful pre-emptive defense strategy against Ebola outbreak in Pakistan.

## Figures and Tables

**Table 1:** Demographic information of respondents.

Demographic Information	Frequency (%)
Gender	
Male	173 (42.92)
Female	230 (57.07)
**Age**	
25–30 years	205 (50.86)
31–35 years	47 (11.66)
36–40 years	36 (8.93)
41–50 years	38 (9.42)
51 and above	77 (19.09)
**Organization**	
Government	223 (55.32)
Private	177 (43.92)
**Designation**	
General practitioner	89 (22.08)
Chief medical officer	54 (13.39)
Head of department	10 (2.48)
Resident medical officer	52 (12.90)
Faculty	198 (49.11)
**Experience**	
Less than 5 years	185 (45.90)
5–10 years	73 (18.11)
10–15 years	39 (9.67)
15–20 years	62 (15.38)
20 and above	44 (10.91)
**Average number of patients per day**	
1–30	114 (55.3)
31–60	68 (33.0)
61–90	20 (9.7)
**Locality of Practice**	
Urban	207 (51.36)
Peri-urban	196 (48.63)

**Table 2:** Cross-tabulation of respondents’ demographics with knowledge.

Demographics	Knowledge Regarding EVD			
Gender	No Knowledge	No Adequate Knowledge	Somewhat Knowledgeable	Adequate Knowledge
Male	15 (23.9)	28 (21)	78 (76.6)	4 (4.4)
Female	50 (41.1)	28 (36)	130 (131.4)	8 (7.6)
**Designation**				
General practitioner	21 (13.3)	10 (14.8)	51 (56.2)	7 (4.7)
Chief medical officer	5 (6)	3 (6.6)	29 (25.3)	3 (2.1)
Head of department	2 (1.5)	1 (1.7)	5 (6.3)	2 (0.5)
Resident medical officer	5 (7.8)	13 (8.6)	33 (32.8)	1 (2.7)
Professor	0 (1.2)	2 (1.3)	6 (5.1)	0 (0.4)
Lecturer	4 (7.2)	12 (8)	32 (30.3)	0 (2.5)
**Experience**				
Less than 5 years	43 (34.3)	32 (29.7)	90 (97.7)	3 (6.3)
Between 5 to 10 years	8 (9.8)	10 (8.5)	28 (27.9)	2 (1.8)
Between 11 to 15 years	6 (3.5)	3 (3)	7 (9.9)	1 (0.6)
Between 16 to 20 years	2 (7.8)	6 (6.7)	24 (22.1)	5 (1.4)
Above 20 years	0 (4.7)	1 (4.1)	22 (13.4)	0 (0.9)

**Table 3:** Respondents’ opinion towards personal protection and management strategies.

Case Management Strategies Available	No. (%)
Supportive treatment	270 (66.93)
Approved vaccine	88 (21.83)
Immunoglobulin	45 (11.16)
**Personal Protection Among Health Care Workers**	
Hand hygiene	31 (7.69)
Personal protective equipment	66 (16.37)
Proper handling of sharps and needles	25 (6.20)
All of them	281 (69.72)
**Personal Protective Equipment Recommended by WHO**	
Double examination gloves	40 (9.92)
Impermeable gown	15 (3.72)
Medical mask/face shield	21 (5.21)
Shoe covers	2 (0.49)
Head cover	1 (0.24)
All of them	324 (80.39)
